# Gut microbiome predicts selenium supplementation efficiency across different Chinese adult cohorts using hybrid modeling and feature refining

**DOI:** 10.3389/fmicb.2023.1291010

**Published:** 2023-10-17

**Authors:** Sikai Jiang, Bailu Zhang, Xiaoqian Fan, Yuzhu Chen, Jian Wang, Shunyao Wu, Lijuan Wang, Xiaoquan Su

**Affiliations:** ^1^College of Computer Science and Technology, Qingdao University, Qingdao, China; ^2^Shouguang Hospital of Traditional Chinese Medicine, Weifang, China; ^3^Maikeruo Medical Technology Co., Ltd., Suzhou, China

**Keywords:** human health, selenium, gut microbiome, precise nutrition, modeling

## Abstract

Selenium (Se) is an essential trace element that plays a vital role in various physiological functions of the human body, despite its small proportion. Due to the inability of the human body to synthesize selenium, there has been increasing concern regarding its nutritional value and adequate intake as a micronutrient. The efficiency of selenium absorption varies depending on individual biochemical characteristics and living environments, underscoring the importance of accurately estimating absorption efficiency to prevent excessive or inadequate intake. As a crucial digestive organ in the human body, gut harbors a complex and diverse microbiome, which has been found to have a significant correlation with the host’s overall health status. To investigate the relationship between the gut microbiome and selenium absorption, a two-month intervention experiment was conducted among Chinese adult cohorts. Results indicated that selenium supplementation had minimal impact on the overall diversity of the gut microbiome but was associated with specific subsets of microorganisms. More importantly, these dynamics exhibited variations across regions and sequencing batches, which complicated the interpretation and utilization of gut microbiome data. To address these challenges, we proposed a hybrid predictive modeling method, utilizing refined gut microbiome features and host variable encoding. This approach accurately predicts individual selenium absorption efficiency by revealing hidden microbial patterns while minimizing differences in sequencing data across batches and regions. These efforts provide new insights into the interaction between micronutrients and the gut microbiome, as well as a promising direction for precise nutrition in the future.

## Background

Trace elements, despite their low concentrations in the human body, play a crucial role in various physiological processes. The recognition of their nutritional value has grown with the improvement of living standards. In recent years, research on trace elements, including iron deficiency (Shi et al., 2023), zinc regulation (Kim and Lee, 2021), and iodine-related issues (Yang et al., 2021), has garnered significant attention. Selenium (Se), one of these trace elements, is essential for human health (Razaghi et al., 2021). Selenium acts as an antioxidant and free radical scavenger, and it plays a vital role in improving the immune system, thus contributing to the prevention and treatment of thyroid disease and cardiovascular disease (Ambra et al., 2023). Since the human body cannot synthesize selenium, external intake through supplementation is necessary (Mojadadi et al., 2021). Typically, dietary sources provide over 20 inorganic and organic selenium compounds, which are absorbed and maintained in the blood through continuous excretion (Kieliszek, 2019). Consequently, moderate daily selenium supplementation is necessary. However, excessive selenium levels can be toxic (Hadrup and Ravn-Haren, 2020). Studies have indicated that improper selenium intake can lead to acute poisoning, characterized by symptoms such as vomiting, diarrhea, pain, and nausea, particularly following excessive oral exposure. Severe toxicity can manifest as cardiovascular and pulmonary symptoms, ultimately resulting in death. Therefore, the recommended daily intake of selenium for adults is 60 μg, with a maximum intake of 400 μg (Kipp et al., 2015).

The absorption efficiency of selenium can vary among individuals, even when ingesting the same amount of selenium. Factors such as age, BMI (body mass index), health status, lifestyle choices (including diet, probiotic intake, and medication use), contribute to this variation (Rayman, 2020). Therefore, the implementation of precise nutrition becomes essential as it enables tailored selenium supplementation programs that quantitatively and accurately meet individual needs, thus preventing selenium deficiency or overload. Currently, selenium levels are primarily determined in human tissues, such as through hair analysis (Li et al., 2020). Consequently, the estimation of selenium absorption efficiency can only be assessed after selenium supplementation intervention. It is crucial to develop methods that can predict the absorption efficiency of selenium in different populations prior to supplementation, which would greatly aid in the design of intervention schemes and the optimization of precision nutrition for micronutrients.

The intestine constitutes the primary digestive organ in the human body, working in conjunction with the gut microbiome to break down intricate fibers and polysaccharides, thereby facilitating the absorption of glucose, vitamins, fats, and trace elements (Mohn and Johnson, 2015). Given its critical role in digestion and immunity, the gut microbiome has emerged as a focal point of research. Comprising thousands of diverse microorganisms, the human gut microbiome interacts with the body and participates in various vital physiological functions, including immune system modulation, metabolism, regulation of the gut-brain axis, as well as nutrient absorption and energy regulation (Marchesi et al., 2016). A growing body of evidence has elucidated the relationship between the composition of the gut microbiome (including taxonomy and function) and various diseases, such as obesity (Annapure and Nair, 2022), inflammatory bowel disease (Hills et al., 2019), colorectal cancer (Rebersek, 2021), mental disorders (Lucas, 2018), etc. The intricate and multifaceted interaction between the gut microbiome and the host presents an opportunity for predicting selenium absorption efficiency in the context of precision nutrition, potentially opening up new avenues for breakthroughs in this field. However, the correlation between selenium absorption efficiency and microbial pattern remains poorly understood.

In this study, we aimed to investigate the feasibility of utilizing the gut microbiome and innovative bioinformatic modeling for achieving precise nutrition in diverse cohorts of Chinese adults. To accomplish this, we employed a hybrid predictive modeling approach that incorporated refined features of the gut microbiome and re-encoded host variables. This approach not only enabled the establishment of a correlation between selenium absorption and the microbiome, but also effectively mitigated the variations in sequencing data arising from different batches and regions. By leveraging this methodology, we can enhance the implementation of personalized nutrition strategies tailored to individual circumstances, thereby reducing the risk of selenium deficiency or overload. Our findings highlight the potential of integrating gut microbiome analysis and hybrid modeling techniques to optimize precision nutrition approaches.

## Results

### Brief information of cohort design and selenium supplementation

**Figure 1:**
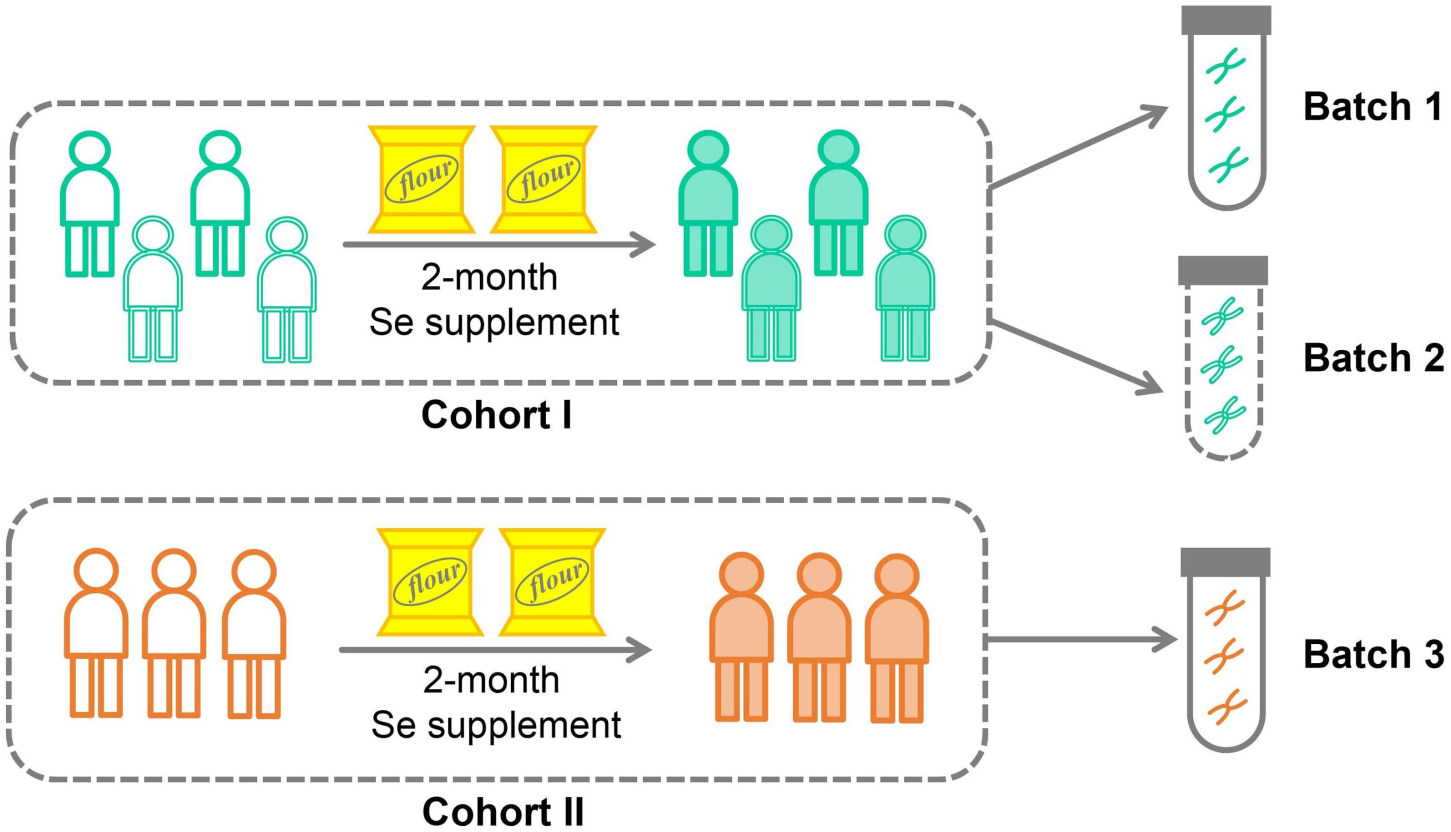
Cohort design for selenium supplementation.

In this study, we conducted a selenium nutritional supplementation intervention involving 266 adult participants from two cities in China (Figure 1). To assess the effectiveness of the intervention, we measured selenium content from hair roots and analyzed the gut microbiome using 16S rRNA gene amplicon sequencing before and after the intervention period. Additionally, we collected comprehensive physiological and clinical metadata, including gender, age, BMI, probiotic supplementation, and disease status. Following data curation, which involved ensuring the completeness of intervention progress and host metadata, 206 subjects were kept in the data analysis. These subjects were divided into two cohorts based on their resident location (Figure 1; Table 1): Cohort I contains 199 subjects recruited from Shijiazhuang City, of which 156 were sequenced by Batch 1, and 43 were sequenced by Batch 2; Cohort II contains 7 subjects recruited from Suzhou City, and they were sequenced by Batch 3. Based on our experience with selenium supplementation and the distribution of samples, we set the threshold for selenium absorption rate at 10% (Hardy et al., 2012). Consequently, all subjects were categorized into two groups: the low-efficiency selenium absorption group (<10%; LE group) and the high-efficiency selenium absorption group (≥10%; HE group). For further details on the experimental design, cohort recruitment, and sequence processing, please refer to the **Methods and Materials** section.

**Table 1:** Brief information of cohorts.

Cohort	Location	# of subjects	16S region	Sequencing batch	Sequencing platform
Cohort I	Shijiazhuang	156	V4	Batch 1	Novaseq 6,000 PE150
Cohort I	Shijiazhuang	43	V4	Batch 2	Novaseq 6,000 PE150
Cohort II	Suzhou	7	V4	Batch 3	Novaseq 6,000 PE150

### Gut microbiome predicts the selenium absorption efficiency by feature re-encoding and integration

**Figure 2:**
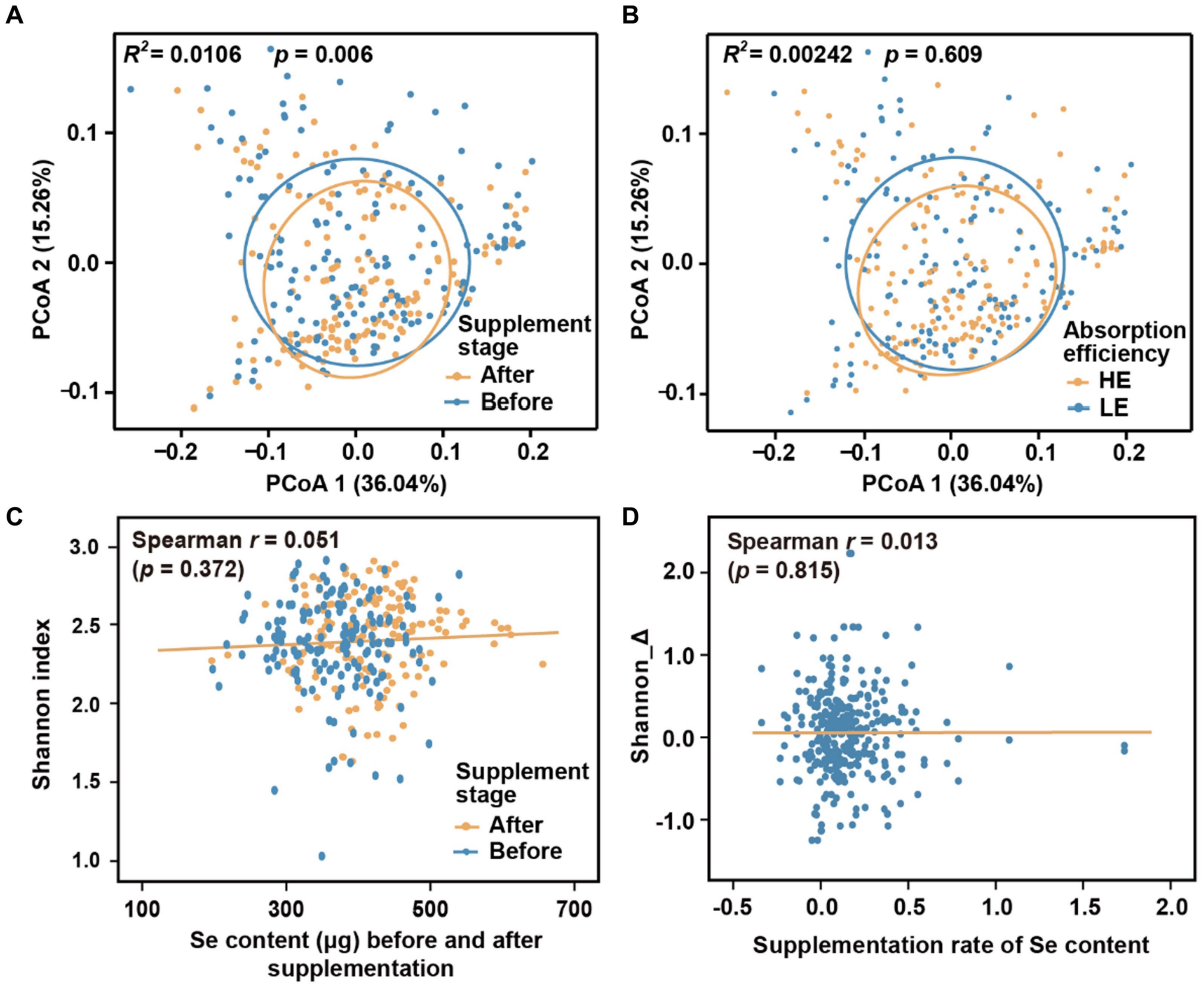
Microbial diversity across supplementation stages and groups. **(A)** Beta-diversity before and after selenium nutrient supplementation intervention. **(B)** Beta-diversity between LE and HE groups. **(C)** Correlation between alpha-diversity and hair root selenium content before and after selenium supplementation. **(D)** Correlation between the change of alpha-diversity and supplementation rate of selenium content. Principal coordinates of beta-diversity were produced by Meta-Storms distances, and alpha-diversity was measured by Shannon index.

We initially investigated the dynamics of the microbial community during different stages of selenium supplementation and among different groups. To minimize potential confounding effects from host region and sequencing batch differences, we only used Batch 1 in this analysis. While there were changes in beta-diversity observed with the progression of selenium intervention (Figure 2A; Adonis *R^2^* = 0.011, *p* = 0.006; *p*-values threshold for statistical significance set as 0.01), no significant differences were observed between the low-efficiency (LE) and high-efficiency (HE) absorption groups (Figure 2B; Adonis *R^2^* = 0.002, *p* = 0.609). Additionally, the low correlation coefficients (|*r*| < 0.1, *p* > 0.01) between the alpha diversity indices (Shannon, Simpson, and Chao1) and hair selenium content (Figures 2C,D; Supplementary Figure S1) further indicated a weak association between the overall gut microbiome and selenium supplementation.

**Figure 3:**
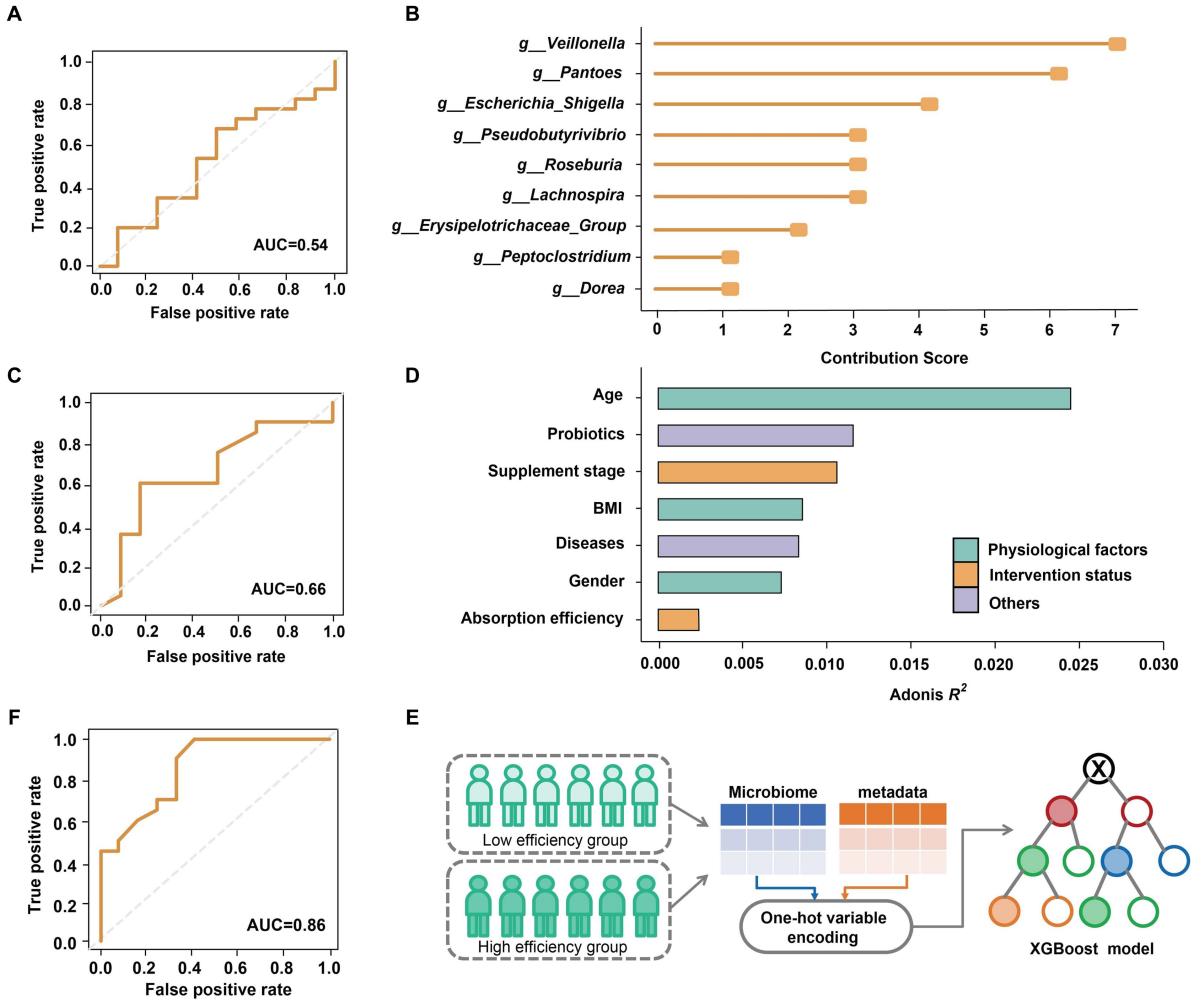
Classification of selenium supplementation efficiency on Batch 1. **(A)** AUC of the model trained from all genus-level bacteria. **(B)** Top ten most contributed genera were used as biomarkers. **(C)** AUC of the model trained by ten biomarkers. **(D)** The effect size of physiological factors, selenium intervention status and other host variables. **(E)** Integrating the host metadata in the prediction model by one-hot variable encoding. **(F)** AUC of the model optimized by refined microbial features and encoded host variables.

We further explored the impact of the gut microbiome on predicting the efficiency (low or high) of selenium absorption prior to supplementation, focusing on Batch 1. Using XGBoost (Chen and Guestrin, 2016), we constructed a predictive model based on the relative abundance of genus-level microbial data (49 genera in total; refer to **Methods and Materials** for details). The performance of the model was evaluated using the area under the receiver operating characteristic curve (AUC). Regrettably, the predictive model yielded a modest AUC of only 0.54 (Figure 3A). This limited performance can be attributed to the non-significant differences observed between the gut microbiomes of the high-efficiency (HE) and low-efficiency (LE) absorption groups (Figure 2B).

While the overall structure of the microbiome appeared to be unaffected by selenium absorption efficiency, a deeper analysis uncovered hidden associations. Initially, all genus-level features were ranked based on their contributions to the decision trees in the XGBoost model. We identified the top 10 features as ‘biomarkers’ (Figure 3B; refer to **Methods and Materials** for details; Supplementary Figure S3) and optimized the model by predicting whether subjects could achieve a supplementation rate of 10% or higher using only these biomarkers. The results demonstrated an improved AUC, albeit only reaching 0.66 (Figure 3C).

Considering that various physiological and lifestyle factors can significantly impact the gut microbiome (Chan et al., 2021), we assessed the influence of different variables on beta-diversity (Figure 3D). Interestingly, we observed that age, probiotic intake, BMI, and gender exerted a stronger effect on the gut microbiome compared to selenium absorption rate. Leveraging this observation, we incorporated host metadata into the predictive model using one-hot variable encoding (Figure 3E; refer to **Methods and Materials** for details). Through these comprehensive efforts, our model achieved an impressive AUC of 0.86 (Figure 3F), indicating the ability to estimate the gut microbial nutrient supplementation efficiency by incorporating host metadata.

### Reduction of batch effect by hybrid modeling

**Figure 4:**
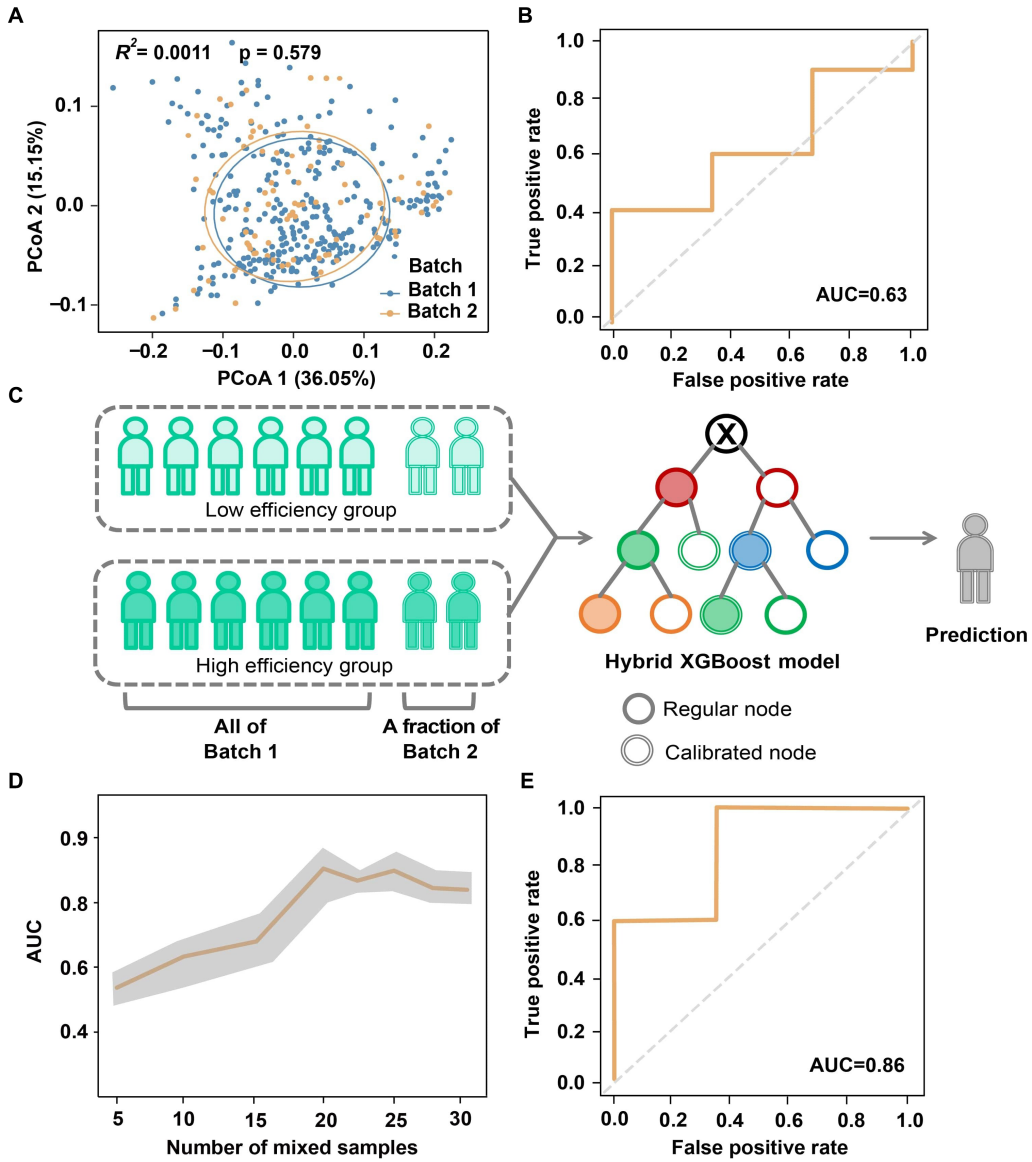
Reduction of batch effect in the classification of absorption efficiency by hybrid modeling. **(A)** Principal coordinates of gut microbiome of two sequencing batches of Cohort I. **(B)** AUC obtained by directly using the model of Batch 1 to Batch 2. **(C)** Reduction of batch effect by hybrid modeling**. (D)** Variation of AUC associated with the number of Batch 2 samples mixed for model calibration. **(E)** The optimal AUC of hybrid modeling.

Due to variable factors during the sequencing process, it is important to address the compatibility of microbiome-based models across different sequencing batches (Chen et al., 2011; Sun et al., 2019). To investigate batch effects within the same region, we compared sequencing samples from Batch 1 and Batch 2 of Cohort I. Both batches showed no significant differences in microbial composition (Adonis *R^2^* = 0.001, *p* = 0.579; Figure 4A). However, when we applied the XGBoost model trained on Batch 1 (Figure 3F) to Batch 2 for testing, the AUC decreased to only 0.63 (Figure 4B). In other words, although the gut microbiome generated by the two batches displayed high similarity, the presence of batch effects still introduced confounding factors that affected the classification and identification of the absorption status.

Here we implemented a hybrid predictive modeling approach to minimize cross-batch and regional sequencing data differences (Figure 4C). Specifically, the prediction model developed on Batch 1 was further calibrated by incorporating samples from Batch 2, which were randomly chose with number ranging from *n* = 5 to 30 (up to 70% of Batch 2). We then randomly selected *n* = 13 (30% of Batch 2) subjects from the remaining Batch 2 as the testing set. To mitigate any potential biases, the modeling and validation procedure was repeated 5 times with different randomizations. As depicted in Figure 4D, we observed a sheer increase in AUC when mixing *n* = 15 for calibration, which then stayed at a relatively stable level after adding *n* = 20 samples. Ultimately, this cross-batch classification approach achieved an optimal AUC of 0.86 (Figure 4E), which is comparable to the AUC obtained from the same data batch.

### Cross-cohort feature refining expanded the classification among regions

**Figure 5:**
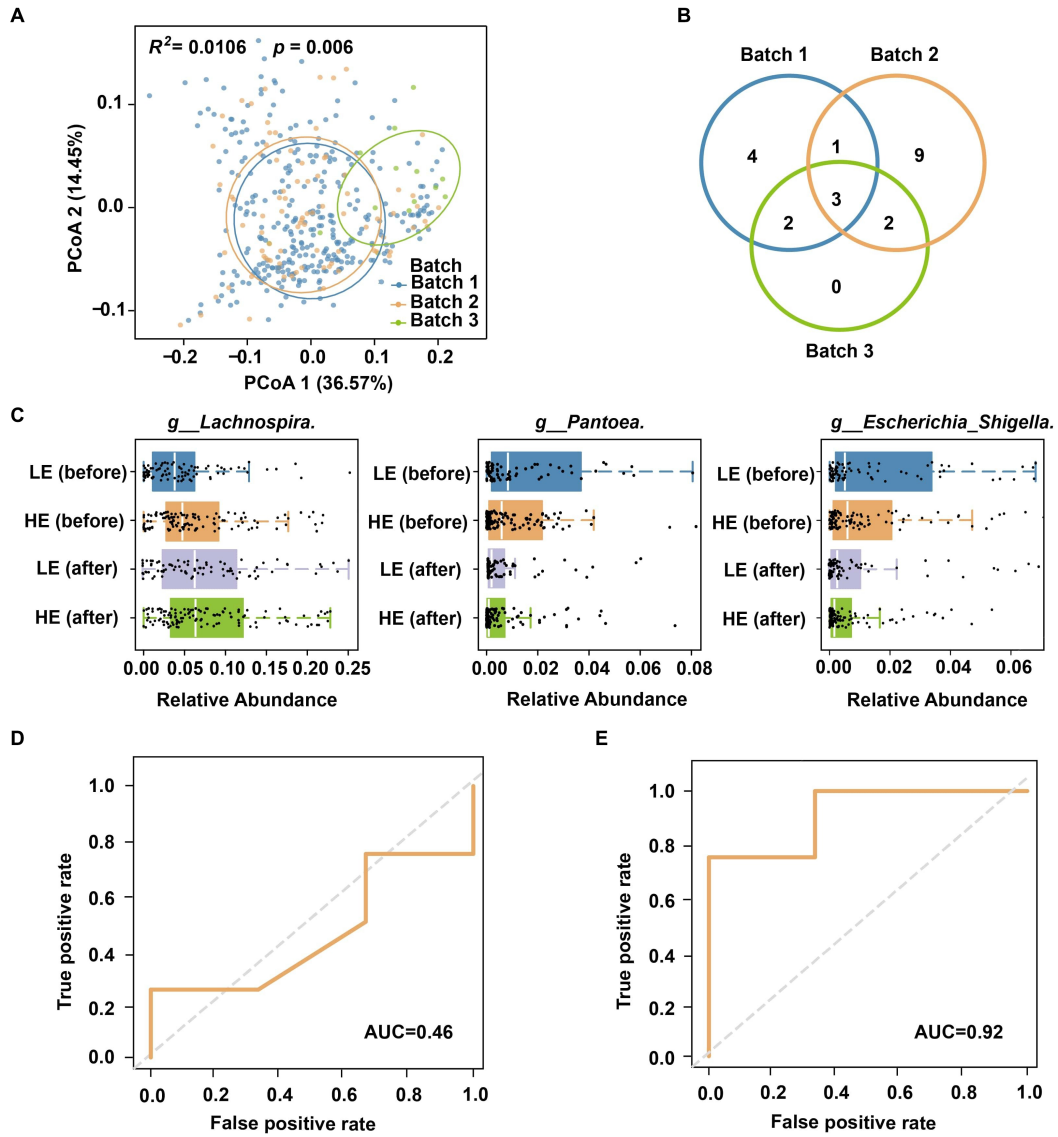
Cross-cohort prediction of selenium supplementation efficiency. **(A)** Principal coordinates of multiple cohorts and sequencing batches. **(B)** Venn plot of biomarkers for the three batches. **(C)** Distribution of biomarkers in different stages and groups. **(D)** AUC was obtained by directly using the model of Cohort I to Cohort II. **(E)** AUC of cross-cohort classification after shared feature screening.

Geographical location is a strong influence factor on human gut microbiome (Sun et al., 2019). Our findings also revealed such a pattern through beta diversity analysis of different batches from two cities (Figure 5A; Adonis *R^2^* = 0.033, *p* = 0.001; Supplementary Figure S2). Therefore, using a model trained on microbiomes from one city may result in reduced sensitivity or accuracy when applied to another city (Sun et al., 2019). For instance, when directly utilizing a model trained on Batch 1 to predict the selenium absorption efficiency of Batch 3, the AUC dropped from 0.86 to 0.46 (Figure 5D). On the other side, a predictive model derived from samples of the same region as the training data is ideal (e.g., a model built by Cohort II for Batch 3). However, implementing this strategy in practical scenarios presents challenges due to sampling and sequencing costs, as well as the limited availability of training samples at each specific location (e.g., there were only 7 samples in Cohort II).

A comprehensive analysis comparing the biomarker classification taxa for selenium supplementation and absorption efficiency across the three batches revealed microbial variations among different regions. The Venn diagram of Figure 5B highlights the presence of specific markers in each batch, indicating the limited applicability of models for cross-regional classification. For example, among the 10 bacterial genera used in Batch 1 model (Figure 3B), some were absent in another batch (Batch 2) or city (Batch 3). However, we noticed that three of them (*g__Lachnospira*, *g__Pantoea*, *g__Escherichia_Shigella*) were region/batch-specific that commonly shared across all batches. The distribution of these shared genera was also significantly associated with the selenium intervention process and magnitude (Figure 5C). Hence, we further refined the model trained on Cohort I by retaining only the three shared microbial features (metadata was still encoded and integrated). Remarkably, this modified model successfully predicted the selenium absorption efficiency of Cohort II, achieving an impressive AUC = 0.92 (Figure 5E). In this way, the process of cross-cohort feature screening and refinement offers a viable strategy to extend the application of the data model to different regions.

## Conclusion and discussion

Achieving the right balance of selenium intake is vital to human health. However, the response to selenium intake can vary among individuals due to their unique physiological conditions. Therefore, a one-size-fits-all approach to selenium supplementation is not optimal for the entire population. In this study, we utilized various strategies such as factor selection, feature refinement, variable encoding, and prediction modeling to establish the gut microbiome as an indicator of selenium absorption efficiency before supplementation begins. This personalized approach to managing individual micronutrient needs contributes to the advancement of precision nutrition.

Furthermore, the influence of lifestyle and physiological variables of the host, as well as biases introduced by sequencing batch effects, can significantly impact the classification and identification of microbiome data. While it is feasible to minimize these confounding factors in well-designed cohorts during scientific studies, their impact cannot be completely avoided in practical scenarios. To address this challenge, we developed a novel machine learning strategy that combines hybrid modeling, feature extraction, and integration of host variables, offering a solution for detecting microbiome data across different batches and cohorts. These approaches also hold promise for future research involving microbiome-based health status prediction and disease diagnosis, thereby paving the way for broader applications of microbiome research.

It is important to note that our study has limitations in terms of sampling region, cohort size and time points, which restricts the generalizability of the findings to specific locations and periods. While our screening method provides valuable insights, employing advanced techniques such as metagenomic high-throughput sequencing on time-series samples could yield more robust and stable results, despite the potential complexity and cost associated with these methods.

## Methods and materials

### Cohort design and sample collection

A total of 266 adult subjects were recruited from two cities in China to participate in this study. Prior to the intervention, all subjects were not undergoing high-selenium intervention. The participants then underwent a 2-month selenium nutrient supplementation intervention, where they were provided with high selenium corn flour at a dosage of 100 μg/day. During the experiment, we controlled certain confounding factors, such as dietary habits, extended cross-regional travel, and hair treatments (dyeing/perming), with the consent of the participants. However, we refrained from implementing strict controls for other factors such as probiotic supplementation and medication use, considering ethical considerations. The study protocol, including all procedures and data collection, was submitted to the ethical review board for approval (refer to **Ethics approval** for details). All necessary informed consent procedures were followed, and the adult participants were investigated in accordance with the approved protocol.

Hairs and feces were collected from each participant at both the initial stage and after the intervention. Hair samples were collected for selenium content measurement, while fecal samples were collected for surveying the gut microbiome. In addition, metadata including gender, age, BMI, probiotics intake, and health status were recorded for each participant (refer to Supplementary Table S1). Age was categorized into three groups: Young (≤35), Middle (36–59), and Old (≥60). BMI was divided into three categories: Low (<18.5), Middle (18.5–23.9), and High (≥24). Participants who failed to follow the selenium supplementation schedule, did not meet the required dosage, lacked paired samples during the selenium supplementation progress, or had insufficient host metadata were excluded from the analysis. After exclusions, a total of 206 subjects were included for the data analysis.

### DNA extraction, sequencing and data processing

Totally 412 fecal samples (206 subjects, 2 stages) were enrolled for DNA extraction and 16S rRNA amplicon sequencing. DNA of each sample was extracted at Hangzhou Guhe Information Technology Co., LTD. using a GUHEF100 kit according to the manufacturer’s recommendations, and then DNA concentrations were quantified using Qubit 2.0 Fluorometer (Invitrogen). The V4 hypervariable region of the 16S rRNA was amplified using the common primers 515F (5′ -GTGCCAGCMGCCGCGGTAA −3′) and 806R (5′- GGACTACHVGGGTWTCTAAT −3′). The purified PCR products were constructed with a short fragment library with a 2 × 150 paired-end (PE) configuration and sequenced using the Novaseq 6,000 platform. Raw sequences were pre-processed by Parallel-Meta Suite (PMS, version 3.7) (Chen et al., 2022), including data quality control, chimera removal, reads double-end merging, and ASV (amplicon sequence variant) denoising. Taxonomy was annotated against GreenGenes database (version 13–8) (DeSantis et al., 2006) by 99% similarity.

### Diversity and statistical analysis of gut microbiome

The Shannon index, Simpson index and Chao1 index of alpha-diversity were calculated using the genus-level profiles (Liu et al., 2021). Statistical significance between groups was measured by the Wilcoxon rank sum test, and the Spearman correlation coefficient was used for correlation analysis. Distances among samples were calculated using weighted Meta-Storms algorithm (Su et al., 2014; Jing et al., 2019), and statistical significance on beta-diversity and effect sizes of host variables were measured by the Adonis analysis (also named as Permutational multivariate analysis of variance, PERMANOVA). We set 0.01 as the threshold of statistical significance for *p*-values. All analytical procedures were performed by Parallel-Meta Suite (version 3.7).

### Machine learning and prediction modeling

We utilized the XGBoost module from Scikit-learn (Pedregosa et al., 2011) to construct our machine learning model. The performance evaluation was based on the area under the receiver operating characteristic curve (AUC). Through a heuristic search (Supplementary Table S2), we fine-tuned the parameters during a 5-fold cross-validation on Batch 1. Initially, we trained an XGBoost model using all features, and obtained weight importance score of each feature in classification. Then all features were ranked in a descending order based on their importance score. After that, we re-built the model by top *n* feature iteratively to perform classification and conducted the learning curve. In the learning curve (Supplementary Figure S3), prediction accuracy stabilized around the *n* = 10 features and experienced a sharp decline thereafter. Consequently, we selected the top 10 ranked features as biomarkers for further modeling.

To incorporate host variables, including gender, age, BMI, probiotics intake, and health status, into the model training, we utilized one-hot coding to integrate them with the microbial features (Falony et al., 2016). Firstly, all host variables were discretized and categorized. Then, each variable was encoded using a binary vector, where each category was represented by a binary bit. For instance, gender was divided into two categories, Male and Female, encoded as ‘(1, 0)’ and ‘(0, 1)’ respectively, occupying two columns in the data table. Similarly, age was divided into three categories, Young (≤35), Middle (36–59), and Old (≥60), encoded as ‘(1, 0, 0)’, ‘(0, 1, 0)’, and ‘(0, 0, 1)’ respectively, and so on Supplementary Table S3. After encoding the metadata, we combined it with microbial characteristics using L2 regularization and normalization techniques to prevent overfitting (Laarhoven, 2017).

## Data availability statement

The datasets presented in this study can be found in online repositories. The names of the repository/repositories and accession number(s) can be found in the article/Supplementary material.

## Ethics statement

The studies involving humans were approved by the Ethics Committee of Jiangsu Rongjun Hospital (approval No. YKT2021002). The studies were conducted in accordance with the local legislation and institutional requirements. The participants provided their written informed consent to participate in this study.

## Author contributions

SJ: Formal analysis, Methodology, Software, Writing – original draft. BZ: Formal analysis, Methodology, Software, Writing – original draft. XF: Conceptualization, Investigation, Writing – review & editing. YC: Investigation, Methodology, Writing – review & editing. JW: Investigation, Writing – review & editing. SW: Data curation, Methodology, Supervision, Writing – review & editing. LW: Conceptualization, Investigation, Supervision, Writing – review & editing. XS: Conceptualization, Funding acquisition, Resources, Supervision, Writing – review & editing.
