# Supplementary material for: Gut microbiome predicts selenium supplementation efficiency across different Chinese adult cohorts using hybrid modeling and feature refining
Source: Front Microbiol. 2023 Oct 17;14:1291010. doi: 10.3389/fmicb.2023.1291010 (PMC10616252; doi:10.3389/fmicb.2023.1291010)
Supplement: Supplementary file 1 [file Table_1.DOCX]

**Supplementary Figures**


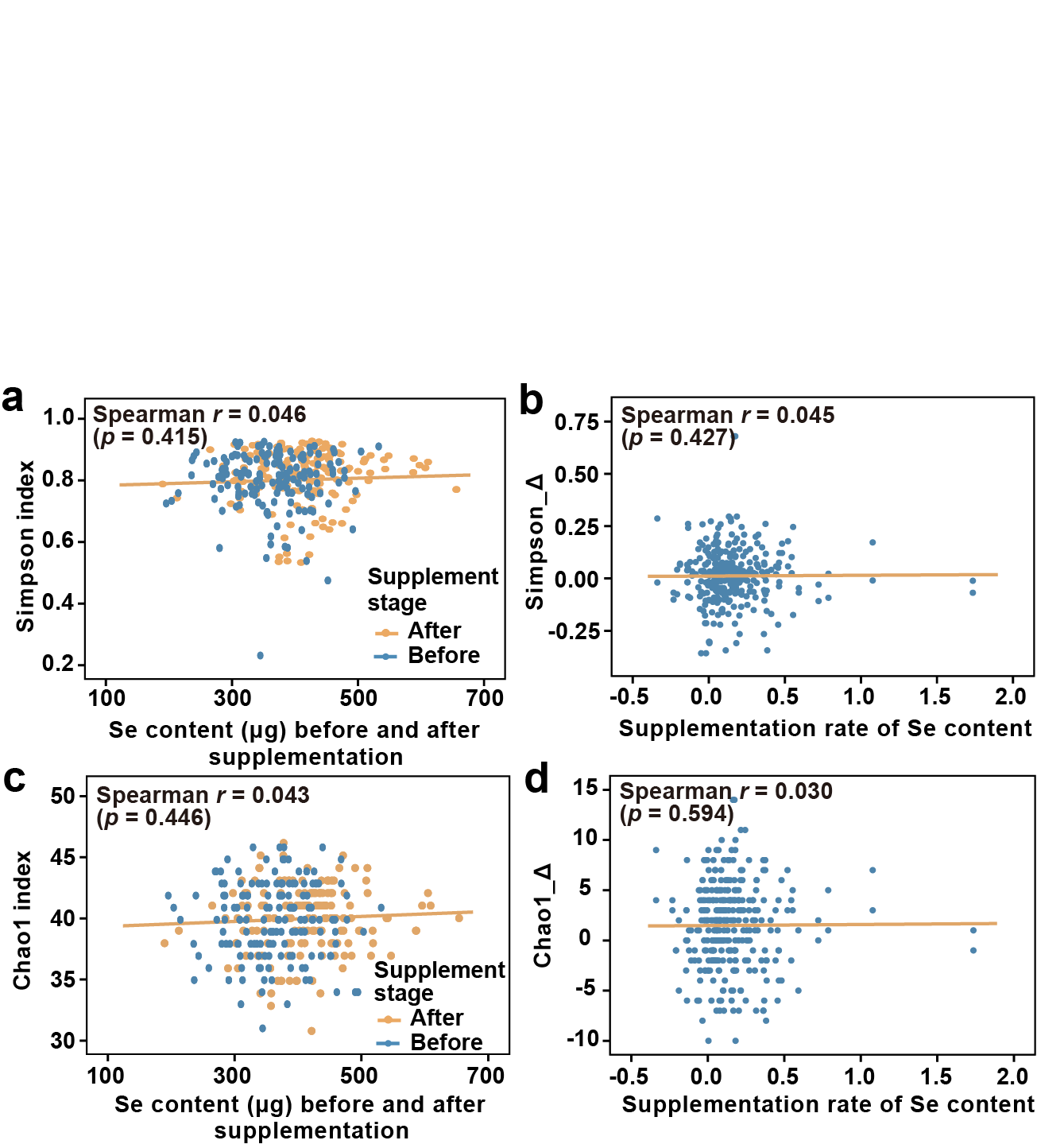


**Fig. S1. Correlation between hair root selenium content and alpha-diversity of Simpson index (a, b) and Chao1 index (c, d) in different stages.**

**
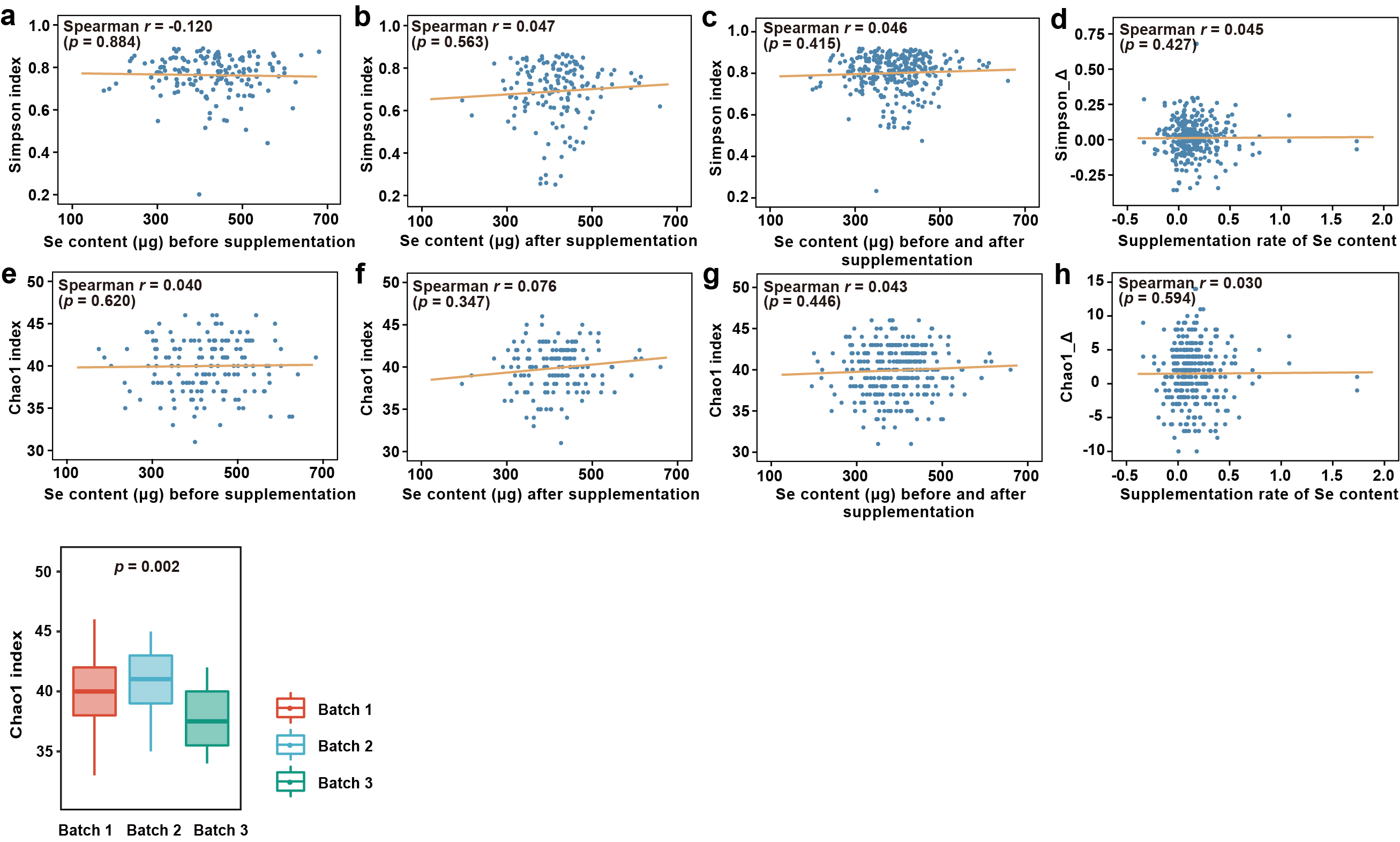
**

**Fig. S2. Distribution of alpha diversity among multiple cohorts and sequencing batches.**


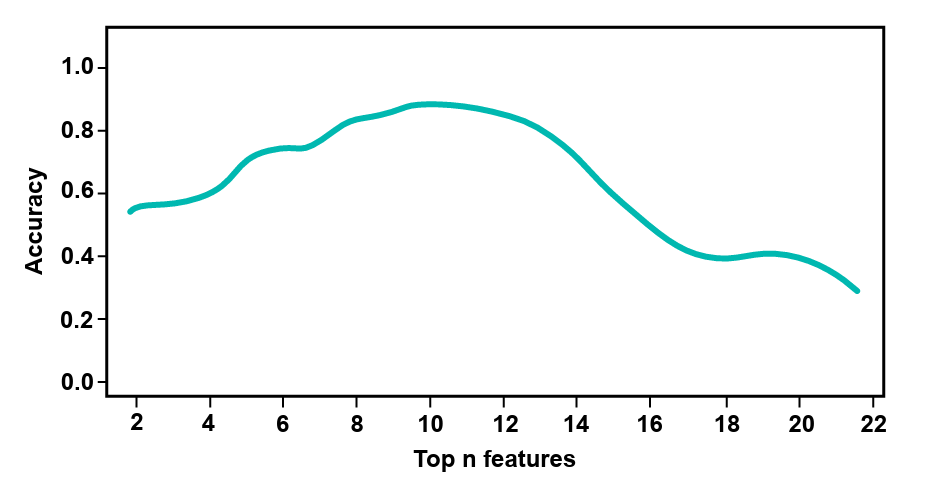


**Fig. S3. The learning curve of the top *n* features.**
